# Ethyl 2-[(4-chloro­phen­yl)hydrazono]-3-oxobutanoate

**DOI:** 10.1107/S1600536809012951

**Published:** 2009-04-10

**Authors:** Hoong-Kun Fun, Suchada Chantrapromma, Mahesh Padaki, Radhika, Arun M. Isloor

**Affiliations:** a X-ray Crystallography Unit, School of Physics, Universiti Sains Malaysia, 11800 USM, Penang, Malaysia; b Crystal Materials Research Unit, Department of Chemistry, Faculty of Science, Prince of Songkla University, Hat-Yai, Songkhla 90112, Thailand; c Department of Chemistry, National Institute of Technology–Karnataka, Surathkal, Mangalore 575 025, India

## Abstract

The mol­ecule of the title oxobutanoate derivative, C_12_H_13_ClN_2_O_3_, is nearly planar; the inter­planar angle between the benzene ring and the mean plane through the hydrazono-3-oxobutanoate unit is 2.69 (3)°. An intra­molecular N—H⋯O hydrogen bond generates an *S*(6) ring motif. In the crystal packing, C—H⋯O(3-oxo) inter­actions link mol­ecules into dimers. The dimers thus formed are linked through C—H⋯O(carboxyl­ate C=O) inter­actions, leading to the formation of ribbons along  the [01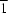] direction, which are stabilized *via* Cl⋯Cl [3.2916 (3) Å] contacts. The ribbons are stacked *via* C⋯O contacts [3.2367 (12)–3.3948 (12) Å].

## Related literature

For hydrogen-bond motifs, see Bernstein *et al.* (1995 ▶). For background to the bioactivity and applications of oxobutanoate derivatives, see: Alpaslan *et al.* (2005 ▶); Billington *et al.* (1979 ▶); Stancho *et al.* (2008 ▶). For the synthesis, see Amir & Agarwal (1997 ▶). For the stability of the temperature controller used in the data collection, see Cosier & Glazer (1986 ▶).
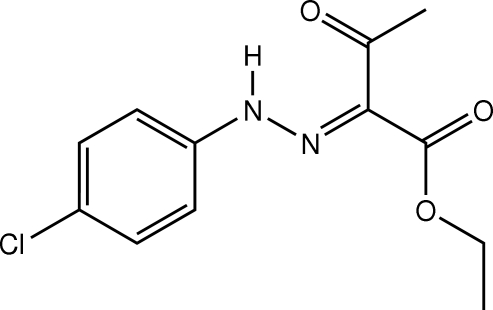


## Experimental

### 

#### Crystal data

C_12_H_13_ClN_2_O_3_
*M* *_r_* = 268.69
Monoclinic, 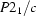

*a* = 4.0259 (1) Å
*b* = 17.0892 (4) Å
*c* = 18.4934 (5) Å
β = 96.802 (1)°
*V* = 1263.38 (6) Å^3^
*Z* = 4
Mo *K*α radiation
μ = 0.30 mm^−1^
*T* = 100 K
0.77 × 0.13 × 0.06 mm

#### Data collection

Bruker APEXII CCD area-detector diffractometer
Absorption correction: multi-scan (*SADABS*; Bruker, 2005 ▶) *T*_min_ = 0.799, *T*_max_ = 0.982
38796 measured reflections
4723 independent reflections
3972 reflections with *I* > 2σ(*I*)
*R*_int_ = 0.039

#### Refinement

*R*[*F* ^2^ > 2σ(*F* ^2^)] = 0.037
*wR*(*F* ^2^) = 0.108
*S* = 1.06
4723 reflections
165 parameters
H-atom parameters constrained
Δρ_max_ = 0.53 e Å^−3^
Δρ_min_ = −0.24 e Å^−3^

### 

Data collection: *APEX2* (Bruker, 2005 ▶); cell refinement: *SAINT* (Bruker, 2005 ▶); data reduction: *SAINT*; program(s) used to solve structure: *SHELXTL* (Sheldrick, 2008 ▶); program(s) used to refine structure: *SHELXTL*; molecular graphics: *SHELXTL*; software used to prepare material for publication: *SHELXTL* and *PLATON* (Spek, 2009 ▶).

## Supplementary Material

Crystal structure: contains datablocks global, I. DOI: 10.1107/S1600536809012951/tk2415sup1.cif

Structure factors: contains datablocks I. DOI: 10.1107/S1600536809012951/tk2415Isup2.hkl

Additional supplementary materials:  crystallographic information; 3D view; checkCIF report

## Figures and Tables

**Table 1:** Hydrogen-bond geometry (Å, °)

*D*—H⋯*A*	*D*—H	H⋯*A*	*D*⋯*A*	*D*—H⋯*A*
N1—H1⋯O1	0.91	1.87	2.5721 (12)	132
C2—H2*A*⋯O2^i^	0.93	2.45	3.3536 (13)	164
C5—H5*A*⋯O1^ii^	0.93	2.53	3.4293 (12)	163

Symmetry codes: (i) 
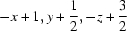
; (ii) 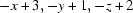.

**Fig. 1.**
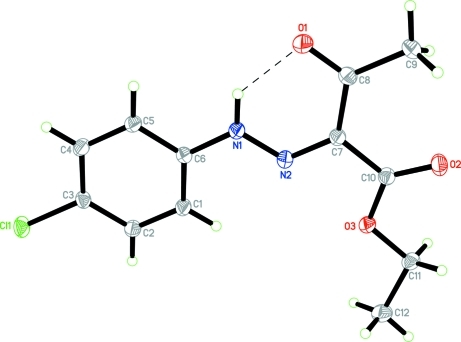
The structure of (I), showing 50% probability displacement ellipsoids and the atom-numbering scheme. The N—H···O hydrogen bond was drawn as a dashed line.

**Fig. 2.**
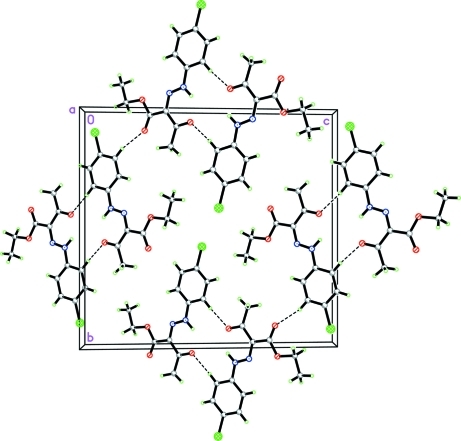
The packing diagram of (I), viewed along in projection the a axis, showing the arrangement of the dimers into molecular ribbons. C—H···O contacts are shown as dashed lines.

## supplementary crystallographic information

### Comment

Derivatives of oxobutanoates are biologically important. 4-Methylthio-2-oxobutanoate was identified in the culture fluids of a range of bacteria, e.g. the yeast *Saccharomyces cerevisiae* and the fungus *Penicillium digitatum* (Billington *et al.*, 1979). Some oxobutanoates exhibit cytotoxic properties (Stancho *et al.*, 2008). The crystal structure of ethyl 4-chloro-2-[2-(2-methoxyphenyl)hydrazono]-3-oxobutanoate has been reported (Alpaslan *et al.*, 2005).

The molecule of the title oxobutanoate derivative, (I), is nearly planar which can be readily indicated by the interplanar angle between the benzene ring and the mean plane through the hydrazono-3-oxobutanoate unit (N1–N2/O1–O3/C7–C10) is 2.69 (3)°, and the C10–C7–C8–C9 torsion angle of 2.81 (15)°. The ethyl group is slightly deviated from the mean plane of the molecule with the torsion angle C10–O3–C11–C12 being 170.6 (9)°. Within the molecule, an intramolecular N1—H1···O1 hydrogen bond generates a S(6) ring motif (Bernstein *et al.*, 1995) (Table 1).

In the crystal packing, the C5—H5A···O1 interactions link two molecules into a dimer (Table 1 and Fig. 2). The dimers are linked together through C2—H2A···O2 interactions to form molecular ribbons along the [011] direction; these ribbons are further stabilized by Cl···Cl [3.2916 (3)Å] contacts. These ribbons are stacked, being connected by C···O [3.2367 (12), 3.3716 (14) and 3.3948 (12)Å] contacts.

### Experimental

Compound (I) was prepared as reported in literature (Amir & Agarwal, 1997). 4-Chloroaniline (1.27 g, 10 mmol) was dissolved in dilute hydrochloric acid (11.0 ml) and cooled to 273 K in an ice bath. To this cold solution, sodium nitrite (1.6 g in 5.0 ml water) was added. The temperature of the reaction mixture was not allowed to raise above 323 K. The resulting diazonium salt solution was then filtered into a cooled solution of ethylacetoacetate (1.7 ml) and sodium acetate (3.5 g) in ethanol (50 ml). The resulting yellow solid was filtered, washed with ice-cold water, dried and recrystallized from methanol. The yield was 1.95 g (81%); *M*.p. 365 K.

### Refinement

All H atoms were placed in calculated positions with d(N—H) = 0.91 Å, d(C—H) = 0.93 Å for aromatic, 0.97 Å for CH_2_ and 0.96 Å for CH_3_ atoms. The *U*_iso_ values were constrained to be 1.5*U*_eq_ of the carrier atom for methyl-H atoms and 1.2*U*_eq_ for the remaining H atoms. A rotating group model was used for the methyl substituents.

### Crystal data

**Table:**

C_12_H_13_ClN_2_O_3_	*F*(000) = 560
*M**_r_* = 268.69	*D*_x_ = 1.413 Mg m^−^^3^
Monoclinic, *P*2_1_/*c*	Melting point: 365 K
Hall symbol: -P 2ybc	Mo *K*α radiation, λ = 0.71073 Å
*a* = 4.0259 (1) Å	Cell parameters from 4723 reflections
*b* = 17.0892 (4) Å	θ = 1.6–32.9°
*c* = 18.4934 (5) Å	µ = 0.30 mm^−^^1^
β = 96.802 (1)°	*T* = 100 K
*V* = 1263.38 (6) Å^3^	Needle, brown
*Z* = 4	0.77 × 0.13 × 0.06 mm

### Data collection

**Table:**

Bruker APEXII CCD area-detector diffractometer	4723 independent reflections
Radiation source: sealed tube	3972 reflections with *I* > 2σ(*I*)
graphite	*R*_int_ = 0.039
φ and ω scans	θ_max_ = 32.9°, θ_min_ = 1.6°
Absorption correction: multi-scan (*SADABS*; Bruker, 2005)	*h* = −6→6
*T*_min_ = 0.799, *T*_max_ = 0.982	*k* = −26→25
38796 measured reflections	*l* = −28→28

### Refinement

**Table:**

Refinement on *F*^2^	Primary atom site location: structure-invariant direct methods
Least-squares matrix: full	Secondary atom site location: difference Fourier map
*R*[*F*^2^ > 2σ(*F*^2^)] = 0.037	Hydrogen site location: inferred from neighbouring sites
*wR*(*F*^2^) = 0.108	H-atom parameters constrained
*S* = 1.06	*w* = 1/[σ^2^(*F*_o_^2^) + (0.0602*P*)^2^ + 0.255*P*] where *P* = (*F*_o_^2^ + 2*F*_c_^2^)/3
4723 reflections	(Δ/σ)_max_ = 0.002
165 parameters	Δρ_max_ = 0.53 e Å^−^^3^
0 restraints	Δρ_min_ = −0.24 e Å^−^^3^

### Special details

**Table:**

Experimental. The crystal was placed in the cold stream of an Oxford Cryosystems Cobra open-flow nitrogen cryostat (Cosier & Glazer, 1986) operating at 100.0 (1) K.
Geometry. All e.s.d.'s (except the e.s.d. in the dihedral angle between two l.s. planes) are estimated using the full covariance matrix. The cell e.s.d.'s are taken into account individually in the estimation of e.s.d.'s in distances, angles and torsion angles; correlations between e.s.d.'s in cell parameters are only used when they are defined by crystal symmetry. An approximate (isotropic) treatment of cell e.s.d.'s is used for estimating e.s.d.'s involving l.s. planes.
Refinement. Refinement of *F*^2^ against ALL reflections. The weighted *R*-factor *wR* and goodness of fit *S* are based on *F*^2^, conventional *R*-factors *R* are based on *F*, with *F* set to zero for negative *F*^2^. The threshold expression of *F*^2^ > σ(*F*^2^) is used only for calculating *R*-factors(gt) *etc*. and is not relevant to the choice of reflections for refinement. *R*-factors based on *F*^2^ are statistically about twice as large as those based on *F*, and *R*- factors based on ALL data will be even larger.

### Fractional atomic coordinates and isotropic or equivalent isotropic displacement parameters (Å^2^)

**Table:**

	*x*	*y*	*z*	*U*_iso_*/*U*_eq_	
Cl1	1.33403 (7)	0.917736 (14)	0.966487 (14)	0.02331 (8)	
O1	1.1283 (2)	0.43897 (5)	0.92388 (4)	0.02458 (17)	
O2	0.4082 (2)	0.41534 (4)	0.74203 (5)	0.02490 (17)	
O3	0.40449 (18)	0.54627 (4)	0.73206 (4)	0.01834 (15)	
N1	1.0478 (2)	0.58595 (5)	0.89744 (5)	0.01680 (16)	
H1	1.1461	0.5484	0.9277	0.020*	
N2	0.8324 (2)	0.56553 (5)	0.84259 (4)	0.01607 (15)	
C1	0.9700 (2)	0.72408 (6)	0.86488 (5)	0.01779 (18)	
H1A	0.8269	0.7109	0.8235	0.021*	
C2	1.0384 (3)	0.80222 (6)	0.88190 (5)	0.01815 (18)	
H2A	0.9413	0.8418	0.8521	0.022*	
C3	1.2535 (2)	0.82023 (5)	0.94400 (5)	0.01668 (17)	
C4	1.4070 (2)	0.76234 (6)	0.98899 (5)	0.01816 (18)	
H4A	1.5530	0.7756	1.0299	0.022*	
C5	1.3393 (2)	0.68425 (6)	0.97197 (5)	0.01727 (17)	
H5A	1.4408	0.6447	1.0013	0.021*	
C6	1.1185 (2)	0.66568 (5)	0.91065 (5)	0.01550 (17)	
C7	0.7628 (2)	0.49093 (5)	0.82808 (5)	0.01602 (17)	
C8	0.9246 (2)	0.42509 (6)	0.86964 (5)	0.01779 (18)	
C9	0.8553 (3)	0.34161 (6)	0.84741 (6)	0.02176 (19)	
H9A	0.9875	0.3074	0.8804	0.033*	
H9B	0.9116	0.3337	0.7989	0.033*	
H9C	0.6223	0.3304	0.8486	0.033*	
C10	0.5100 (2)	0.47889 (6)	0.76369 (5)	0.01646 (17)	
C11	0.1696 (2)	0.53891 (6)	0.66655 (5)	0.01926 (18)	
H11A	−0.0131	0.5044	0.6752	0.023*	
H11B	0.2804	0.5174	0.6272	0.023*	
C12	0.0397 (3)	0.61975 (7)	0.64728 (6)	0.0245 (2)	
H12A	−0.1123	0.6175	0.6032	0.037*	
H12B	0.2235	0.6536	0.6403	0.037*	
H12C	−0.0749	0.6397	0.6861	0.037*	

### Atomic displacement parameters (Å^2^)

**Table:**

	*U*^11^	*U*^22^	*U*^33^	*U*^12^	*U*^13^	*U*^23^
Cl1	0.03064 (14)	0.01473 (12)	0.02328 (13)	−0.00497 (9)	−0.00211 (9)	−0.00104 (8)
O1	0.0277 (4)	0.0185 (3)	0.0251 (4)	0.0003 (3)	−0.0070 (3)	0.0031 (3)
O2	0.0306 (4)	0.0165 (3)	0.0254 (4)	−0.0037 (3)	−0.0057 (3)	−0.0015 (3)
O3	0.0185 (3)	0.0163 (3)	0.0190 (3)	−0.0001 (2)	−0.0030 (2)	0.0012 (2)
N1	0.0185 (4)	0.0146 (3)	0.0163 (4)	−0.0003 (3)	−0.0018 (3)	0.0001 (3)
N2	0.0163 (3)	0.0162 (3)	0.0156 (3)	−0.0012 (3)	0.0012 (3)	−0.0015 (3)
C1	0.0200 (4)	0.0162 (4)	0.0162 (4)	−0.0009 (3)	−0.0022 (3)	0.0005 (3)
C2	0.0203 (4)	0.0157 (4)	0.0176 (4)	−0.0005 (3)	−0.0011 (3)	0.0010 (3)
C3	0.0191 (4)	0.0137 (4)	0.0170 (4)	−0.0018 (3)	0.0014 (3)	−0.0006 (3)
C4	0.0187 (4)	0.0179 (4)	0.0169 (4)	−0.0016 (3)	−0.0023 (3)	0.0007 (3)
C5	0.0178 (4)	0.0163 (4)	0.0169 (4)	0.0000 (3)	−0.0014 (3)	0.0011 (3)
C6	0.0163 (4)	0.0135 (4)	0.0165 (4)	−0.0005 (3)	0.0012 (3)	−0.0003 (3)
C7	0.0168 (4)	0.0147 (4)	0.0162 (4)	−0.0008 (3)	0.0002 (3)	0.0008 (3)
C8	0.0189 (4)	0.0155 (4)	0.0189 (4)	−0.0003 (3)	0.0019 (3)	0.0015 (3)
C9	0.0250 (5)	0.0149 (4)	0.0248 (5)	0.0003 (4)	0.0001 (4)	0.0017 (3)
C10	0.0170 (4)	0.0162 (4)	0.0160 (4)	−0.0005 (3)	0.0015 (3)	0.0001 (3)
C11	0.0186 (4)	0.0216 (4)	0.0169 (4)	0.0008 (3)	−0.0012 (3)	0.0006 (3)
C12	0.0238 (5)	0.0235 (5)	0.0253 (5)	0.0032 (4)	−0.0016 (4)	0.0039 (4)

### Geometric parameters (Å, °)

**Table:**

Cl1—C3	1.7386 (9)	C4—H4A	0.9300
O1—C8	1.2412 (12)	C5—C6	1.3924 (13)
O2—C10	1.2121 (12)	C5—H5A	0.9300
O3—C10	1.3378 (12)	C7—C8	1.4703 (13)
O3—C11	1.4514 (11)	C7—C10	1.4864 (13)
N1—N2	1.3018 (11)	C8—C9	1.5017 (14)
N1—C6	1.4072 (12)	C9—H9A	0.9600
N1—H1	0.9104	C9—H9B	0.9600
N2—C7	1.3258 (12)	C9—H9C	0.9600
C1—C2	1.3918 (14)	C11—C12	1.5049 (15)
C1—C6	1.3964 (13)	C11—H11A	0.9700
C1—H1A	0.9300	C11—H11B	0.9700
C2—C3	1.3885 (13)	C12—H12A	0.9600
C2—H2A	0.9300	C12—H12B	0.9600
C3—C4	1.3895 (13)	C12—H12C	0.9600
C4—C5	1.3906 (14)		
			
C10—O3—C11	115.60 (8)	C8—C7—C10	122.13 (8)
N2—N1—C6	119.81 (8)	O1—C8—C7	119.05 (9)
N2—N1—H1	119.4	O1—C8—C9	119.11 (9)
C6—N1—H1	120.7	C7—C8—C9	121.82 (9)
N1—N2—C7	121.37 (8)	C8—C9—H9A	109.5
C2—C1—C6	119.32 (9)	C8—C9—H9B	109.5
C2—C1—H1A	120.3	H9A—C9—H9B	109.5
C6—C1—H1A	120.3	C8—C9—H9C	109.5
C3—C2—C1	119.14 (9)	H9A—C9—H9C	109.5
C3—C2—H2A	120.4	H9B—C9—H9C	109.5
C1—C2—H2A	120.4	O2—C10—O3	123.32 (9)
C2—C3—C4	121.80 (9)	O2—C10—C7	124.16 (9)
C2—C3—Cl1	119.38 (7)	O3—C10—C7	112.52 (8)
C4—C3—Cl1	118.82 (7)	O3—C11—C12	106.97 (8)
C3—C4—C5	119.11 (9)	O3—C11—H11A	110.3
C3—C4—H4A	120.4	C12—C11—H11A	110.3
C5—C4—H4A	120.4	O3—C11—H11B	110.3
C4—C5—C6	119.48 (9)	C12—C11—H11B	110.3
C4—C5—H5A	120.3	H11A—C11—H11B	108.6
C6—C5—H5A	120.3	C11—C12—H12A	109.5
C5—C6—C1	121.12 (9)	C11—C12—H12B	109.5
C5—C6—N1	117.30 (8)	H12A—C12—H12B	109.5
C1—C6—N1	121.57 (8)	C11—C12—H12C	109.5
N2—C7—C8	124.07 (8)	H12A—C12—H12C	109.5
N2—C7—C10	113.78 (8)	H12B—C12—H12C	109.5
			
C6—N1—N2—C7	179.20 (9)	N1—N2—C7—C8	−2.05 (15)
C6—C1—C2—C3	0.13 (15)	N1—N2—C7—C10	179.71 (9)
C1—C2—C3—C4	1.12 (16)	N2—C7—C8—O1	3.50 (16)
C1—C2—C3—Cl1	−178.83 (8)	C10—C7—C8—O1	−178.40 (10)
C2—C3—C4—C5	−1.00 (15)	N2—C7—C8—C9	−175.28 (10)
Cl1—C3—C4—C5	178.94 (8)	C10—C7—C8—C9	2.81 (15)
C3—C4—C5—C6	−0.36 (15)	C11—O3—C10—O2	−3.29 (14)
C4—C5—C6—C1	1.61 (15)	C11—O3—C10—C7	177.01 (8)
C4—C5—C6—N1	−177.46 (9)	N2—C7—C10—O2	−178.82 (10)
C2—C1—C6—C5	−1.49 (15)	C8—C7—C10—O2	2.90 (16)
C2—C1—C6—N1	177.54 (9)	N2—C7—C10—O3	0.88 (12)
N2—N1—C6—C5	177.14 (9)	C8—C7—C10—O3	−177.40 (9)
N2—N1—C6—C1	−1.93 (15)	C10—O3—C11—C12	170.62 (9)

### Hydrogen-bond geometry (Å, °)

**Table:**

*D*—H···*A*	*D*—H	H···*A*	*D*···*A*	*D*—H···*A*
N1—H1···O1	0.91	1.87	2.5721 (12)	132
C2—H2A···O2^i^	0.93	2.45	3.3536 (13)	164
C5—H5A···O1^ii^	0.93	2.53	3.4293 (12)	163

Symmetry codes:  (i) −*x*+1, *y*+1/2, −*z*+3/2; (ii) −*x*+3, −*y*+1, −*z*+2.
